# Oral Mycobiome Alterations in Postmenopausal Women: Links to Inflammation, Xerostomia, and Systemic Health

**DOI:** 10.3390/biomedicines12112569

**Published:** 2024-11-09

**Authors:** Claudia Florina Bogdan-Andreescu, Andreea-Mariana Bănățeanu, Cristina-Crenguţa Albu, Cristian-Viorel Poalelungi, Oana Botoacă, Constantin Marian Damian, Laurențiu Mihai Dȋră, Ştefan-Dimitrie Albu, Matei Georgian Brăila, Emin Cadar, Anca Daniela Brăila

**Affiliations:** 1 Department of Speciality Disciplines, Faculty of Dental Medicine, “Titu Maiorescu” University, 031593 Bucharest, Romania; claudia.andreescu@prof.utm.ro (C.F.B.-A.); andreea.banateanu@prof.utm.ro (A.-M.B.); oana.botoaca@prof.utm.ro (O.B.); 2 Department of Genetics, Faculty of Dentistry, “Carol Davila” University of Medicine and Pharmacy, 020021 Bucharest, Romania; 3 Department of Obstetrics and Gynecology, Faculty of Medicine, “Carol Davila” University of Medicine and Pharmacy, 020021 Bucharest, Romania; 4 Department of Obstetrics and Gynecology, University of Medicine and Pharmacy of Craiova, 200349 Craiova, Romania; constantin.damian@umfcv.ro (C.M.D.); laurentiu.dira@umfcv.ro (L.M.D.); anca.braila@umfcv.ro (A.D.B.); 5 Department of Periodontology, Faculty of Dentistry, “Carol Davila” University of Medicine and Pharmacy, 020021 Bucharest, Romania; stefan-dimitrie.albu@drd.umfcd.ro; 6 Faculty of Medicine, “Carol Davila” University of Medicine and Pharmacy, 020021 Bucharest, Romania; matei-georgian.braila2022@stud.umfcd.ro; 7 Faculty of Pharmacy, “Ovidius” University, 900470 Constanta, Romania; emin.cadar@365.univ-ovidius.ro

**Keywords:** postmenopausal women, hormonal changes, medication, xerostomia, oral mycobiome, inflammation

## Abstract

The oral mycobiome plays a critical role in maintaining oral and systemic health, with its composition and function influenced by various physiological and environmental factors. This descriptive review explores the changes in the oral mycobiome among postmenopausal women, examining how aging and associated inflammatory processes contribute to these alterations. These changes are linked to an increased prevalence of xerostomia, oral dysbiosis, and inflammation, which can negatively impact both oral and systemic health. We discuss the impact of hormonal fluctuations and immune senescence on fungal diversity and abundance, highlighting key species implicated in oral and systemic diseases. The review also examines the role of systemic conditions and medications, which are common in postmenopausal women, in further exacerbating oral mycobiome alterations. Lastly, it highlights the need for future research to better understand these interactions and develop targeted therapeutic strategies. The current literature indicates a significant association between menopausal status, age-related mycobiome shifts, and increased inflammatory responses, suggesting potential pathways for intervention.

## 1. Introduction

The oral cavity hosts a diverse range of fungal communities, collectively known as the oral mycobiome. Recent studies have highlighted the significance of fungi within the oral environment and their complex interactions with bacterial communities (bacteriome) in maintaining oral health [1,2,3]. The core oral mycobiome primarily includes *Candida* species, which are the most prevalent, followed by genera such as *Cladosporium*, *Aureobasidium*, and members of the *Saccharomycetales* order [4]. Additionally, less common fungi like *Aspergillus*, *Fusarium*, and *Cryptococcus* have been identified, though in lower proportions [5].

Advances in molecular technologies have expanded our understanding of the oral mycobiome, with up to 139 fungal species identified in the oral cavity [6], where *Candida* remains the dominant genus [7]. Some fungal species are associated with health, while others are linked to disease states [8]. However, a definitive fungal “footprint” correlating specific fungal communities with diseases such as oral cancer [9] or other conditions [10,11] has yet to be established.

The concept of dysbiosis is central to the current understanding of the relationship between the mycobiome and disease. In this context, studies have identified *Candida* and *Malassezia* as significant biomarkers for oral diseases within the salivary mycobiome [12].

The oral mycobiome profile also alters with age. For instance, in infants, the mycobiome is highly variable and bears similarities to the vaginal mycobiome during the first month of life [13,14]. Conversely, in older populations, particularly among denture wearers, there is an increased abundance of *Candida albicans* [15,16].

Various factors, including the use of antibiotics, antifungals, steroids, and chemotherapeutic agents, as well as the presence of oral and systemic diseases, contribute to changes in the oral mycobiome [17,18,19,20,21,22,23]. Despite growing interest, the impact of hormonal changes on the oral microbiome, particularly in relation to menopause, remains understudied.

Menopause, intrinsically linked to aging, brings about significant hormonal changes, leaving postmenopausal women more vulnerable to comorbidities. Increasing evidence suggests that the transition from perimenopause to the postmenopausal stage is associated with chronic systemic inflammation [24]. On a hormonal level, menopause is characterized by a decrease in the production of estrogen and progesterone, with the substantial decline in estrogen being primarily responsible for most menopausal symptoms.

Low levels of estrogen impact the mucous membranes of the mouth similarly to the vaginal mucosa due to the presence of estrogen receptors in both tissues. A reduction in estrogen affects the maturation of the oral mucosa, leading to atrophy and thinning, which increases its sensitivity and makes it more susceptible to mechanical injuries [25,26]. Additionally, hormonal changes influence the salivary glands, due to the presence of estrogen receptors, resulting in alterations in saliva secretion and consistency [25,26].

In the oral cavity, estrogens influence the cytodifferentiation of stratified squamous epithelium and the synthesis and maintenance of fibrous collagen, while progesterone plays a critical role in the balance between bone resorption and formation [27]. High estrogen levels during pregnancy are known to promote gingivitis, whereas low estrogen levels during menopause increase susceptibility to temporomandibular joint degeneration and alveolar bone loss [11]. Estrogen deficiency has been associated with alterations in both oral and gut microbiota [28], yet the specific modifications of the oral mycobiome remain less understood.

This review aims to synthesize the current knowledge on how the oral mycobiome changes in postmenopausal women, with a particular focus on its associations with aging and inflammatory processes.

To fulfill the purpose of our study, we searched and selected from the internet (PubMed, Google Scholar) the most recent publications in the field from the last five years that studied the oral mycobiome in postmenopausal women. The keywords were “menopause” and “oral mycobiome”. The exclusion criteria disregarded other human mycobiome studies except oral, oral medicines, and animal mycobiome studies.

## 2. Hormonal Changes in Postmenopausal Women and Their Impact on the Oral Mycobiome

The decline in estrogen levels during menopause profoundly affects the oral mycobiome, significantly altering the composition and behavior of fungal species, thereby contributing to dysbiosis and increased susceptibility to oral infections [28].

Estrogen plays a crucial role in maintaining the integrity of the oral mucosa by supporting the production of antimicrobial peptides and maintaining mucosal immunity [29].

The reduction in estrogen during menopause weakens these defenses, creating an environment that favors the overgrowth of opportunistic fungal species [30]. Among these, *Candida albicans* is the most prominent, known for its ability to transition from a harmless commensal organism to a pathogenic state under conditions of immune suppression or hormonal imbalance [31,32]. The reduction in estrogen not only impairs mucosal barrier function but also decreases the production of protective factors such as histatins, lysozyme, and lactoferrin, leading to a marked increase in *Candida albicans* colonization and the risk of developing oral candidiasis [29,33].

Beyond *Candida albicans*, other non-albicans *Candida* species, such as *Candida glabrata* and *Candida krusei*, also become more prevalent in the postmenopausal oral cavity [34]. *Candida glabrata*, which is known for its ability to persist in hostile environments, can flourish in the estrogen-deprived conditions of menopause, often leading to persistent infections that are difficult to treat [35]. Similarly, *Candida krusei* can take advantage of hormonal shifts to establish itself as a significant oral pathogen in this population [36].

For a long time, it was widely believed that *Candida* species were the main opportunistic pathogens in the oral cavity, despite occasional isolations of other fungi, particularly in medically compromised patients. These included species like *Saccharomyces cerevisiae*, *Penicillium*, *Aspergillus*, and *Geotrichum* [37].

Next-generation sequencing (NGS) platforms, including Illumina and 454 pyrosequencing, and, more recently, third-generation technologies such as Oxford Nanopore and PacBio have revolutionized fungal diagnostics by enabling the detection of difficult-to-culture and rare pathogens directly from clinical samples [38,39]. Recent NGS studies have identified *Malassezia* species in oral samples [40]. Typically associated with skin conditions, *Malassezia* species have been detected in the oral mycobiome of postmenopausal women, suggesting that hormonal changes may disrupt lipid metabolism, creating a niche for these fungi in the oral cavity [41]. NGS also offers deeper insights into microbial community composition and can identify antimicrobial resistance profiles. Notably, NGS has identified *Malassezia* spp. as a distinctive biomarker, aiding in the differentiation of patients with oral squamous cell carcinoma from healthy individuals [40].

*Aspergillus* species, although less common in the oral environment, can become opportunistic pathogens in postmenopausal women, particularly those with compromised immune systems. The decline in estrogen may reduce the effectiveness of mucosal immune responses, allowing *Aspergillus* to colonize and potentially lead to conditions such as oral aspergillosis [28,37]. Additionally, *Cryptococcus neoformans*, an encapsulated yeast typically associated with systemic infections, can also be found in the oral cavity, particularly in immunocompromised patients, although such occurrences are rare [42].

In addition to the well-documented influence of hormonal changes on *Candida* species, other fungal genera such as *Cladosporium*, *Aureobasidium*, *Saccharomycetales*, *Fusarium*, and *Cryptococcus* also play significant roles in the oral mycobiome of postmenopausal women [43,44]. These fungi, although often present as commensals, can become opportunistic pathogens under the altered hormonal conditions associated with menopause. For instance, *Cladosporium*, typically a common airborne fungus, has been found in oral cavities, where it can cause allergic reactions or exacerbate respiratory conditions, particularly in individuals with weakened mucosal barriers [45]. *Aureobasidium*, another genus commonly found in damp environments, has been isolated from oral samples and is associated with biofilm formation, which can complicate oral hygiene and lead to persistent infections [46].

*Saccharomycetales*, a broad class that includes many yeast-like fungi, can also proliferate in the estrogen-deprived oral environment. These fungi are adept at surviving in nutrient-poor conditions and can contribute to dysbiosis by outcompeting beneficial microbial flora [43].

*Fusarium*, a genus often associated with plant pathogens, can cause opportunistic infections in humans, particularly in the oral cavity where it may exploit weakened immune defenses. These infections can be particularly aggressive, leading to complications that are challenging to treat due to the intrinsic resistance of *Fusarium* to many antifungal agents [47,48]. Finally, *Cryptococcus*, although more commonly associated with systemic infections such as cryptococcal meningitis, can be found in the oral cavity, especially in immunosuppressive patients [49]. The presence of *Cryptococcus* in the oral mycobiome highlights the potential for systemic fungi to colonize and cause disease in the oral environment under certain conditions, particularly when local or systemic immune defenses are compromised [49].

The impact of hormonal changes on these fungal species underscores the importance of understanding the broader spectrum of the oral mycobiome, beyond the more commonly studied *Candida* species. These fungi, while often benign, can become significant pathogens in the altered hormonal landscape of postmenopausal women, leading to an increased risk of oral and systemic fungal infections [43]. The mechanisms by which these fungi adapt to the estrogen-deprived environment and the subsequent effects on host–pathogen interactions are areas that require further research to develop effective strategies for managing fungal dysbiosis in this population.

The characteristics of the oral mycobiome, including specific fungal species, their traits, the impact of aging and hormonal changes, mechanisms of dysbiosis, and the consequences of these changes in postmenopausal women, particularly in relation to aging and inflammation, are presented in Table 1.

The impact of xerostomia in the elderly is profound, leading to difficulties in mastication, swallowing, and speaking, as well as an increased risk of dental caries, oral infections, and complications associated with dentures and orthodontic dental appliances [50,51]. Given the significant impact of reduced salivary secretion on the health and well-being of older adults, it is essential to monitor and manage this condition proactively, particularly through the careful review of medications and the use of saliva substitutes or stimulants [52,53]. Understanding the multifaceted influences on salivary secretion in the elderly, including physiological aging, medication use, and systemic health conditions, is crucial for developing effective management strategies to mitigate the adverse effects of xerostomia in this vulnerable population [54].

**Table 1:** Impact of aging and hormonal changes on fungal species in oral mycobiome and mechanisms of dysbiosis.

Fungal Species	Characteristics	Impact of Aging/Hormonal Changes	Mechanism of Dysbiosis	Consequences of Dysbiosis	References
*Candida albicans*	Forms hyphae; most common oral fungal pathogens	Reduced salivary flow and hormonal changes in postmenopausal women and elderly individuals increase colonization and persistence.	Estrogen decline weakens mucosal immunity, promotes biofilm formation on oral surfaces, increases adhesion and tissue invasion, and enhances extracellular enzyme production (phospholipase, proteinase).	Oral candidiasis, biofilm formation, chronic inflammation, mucosal irritation.	Talapko et al., 2021; David et al., 2023; Samaranayake et al., 2001 [31,32,33]
*Candida glabrata*	Does not form true hyphae; more resistant to antifungals, particularly azoles	Increased colonization in low-estrogen environments such as in postmenopausal women, leading to persistent oral candidiasis.	Hormonal imbalances create a niche for this species, particularly in cases where *C. albicans* is not predominant. Exhibits higher resistance to antifungal agents, especially in compromised immune environment.	Persistent oral candidiasis, especially in immunocompromised individuals.	Fidel et al., 1999; Hassan et al., 2021; Jafarzadeh et al., 2023 [34,35,55]
*Candida krusei*	Naturally resistant to fluconazole; emerging oral pathogen	More frequently isolated in postmenopausal women due to hormonal imbalances that support its growth.	Estrogen decline alters the oral environment, allowing *C. krusei* to thrive and contribute to oral candidiasis. Displays natural resistance to fluconazole, complicating treatment in immunocompromised patients.	Oral candidiasis, particularly in postmenopausal women.	Gómez-Gaviria et al., 2020 [36]
*Candida tropicalis*	Opportunistic pathogen; thrives in immunocompromised individuals	Increased prevalence in postmenopausal women and elderly due to disrupted oral mucosal immunity.	Estrogen decline and aging weaken mucosal defenses, allowing *C. tropicalis* to contribute to dysbiosis. It is highly invasive and associated with higher mortality in immunocompromised individuals.	Oral infections, contribution to dysbiosis.	Ghrenassia et al., 2019 [56]
*Malassezia species*	Traditionally associated with skin conditions; now recognized in oral cavity	Estrogen decline during menopause may alter lipid metabolism, creating conditions favorable for *Malassezia* colonization.	Changes in sebaceous gland function and lipid metabolism contribute to colonization and potential pathogenicity. Involvement in altered immune responses due to hormone-driven changes in lipid production.	Potential role in oral dysbiosis, still under investigation; biomarker for oral squamous cell carcinoma.	Naik et al., 2024; Hobi et al., 2022 [40,41]
*Aspergillus fumigatus*	Environmental mold; can cause opportunistic infections	More common in postmenopausal and elderly women with compromised immune systems, leading to oral aspergillosis.	Weakened immune defenses due to estrogen decline and aging allow *Aspergillus* species to colonize and cause infections. Production of mycotoxins and ability to grow in wide range of conditions exacerbate its pathogenicity.	Oral aspergillosis, rare but severe infections.	Vieira et al., 2017; Deepa et al., 2014 [28,37]
*Cryptococcus neoformans*	Encapsulated yeast; more commonly causes systemic infections	Potential for oral colonization in immunocompromised postmenopausal women due to immune alterations from estrogen decline. Autophagy regulates fungal virulence and sexual reproduction in *Cryptococcus neoformans*	Estrogen decline may weaken immune defenses, allowing this typically systemic pathogen to colonize oral cavity. Its capsule enhances survival in hostile environments, including oral cavity.	Potential oral colonization, cryptococcosis in immunocompromised individuals.	Fleming et al., 2024; Mada et al., 2024; Ripszky Totan et al., 2022; Jiang et al., 2020 [42,49,57,58]
*Candida dubliniensis*	Similar to *C. albicans* but less virulent	Increased prevalence in elderly with low BMI and reduced salivary flow.	Aging-associated xerostomia and immune decline create favorable conditions for colonization. Exhibits biofilm formation and resistance to oxidative stress, enhancing its survival in the oral cavity.	Associated with dental caries.	Defta et al., 2023 [8]
*Saccharomyces cerevisiae*	Used in food production; can be an opportunistic pathogen	Increased oral carriage in institutionalized elderly individuals with poor oral hygiene.	Shared utensils and compromised immunity in institutional settings lead to cross-contamination and colonization. Produces ethanol and acetaldehyde, which can lead to local tissue damage and inflammation.	Disruption of normal oral flora, possible infection.	Deepa et al., 2014 [37]
*Aspergillus niger*	Environmental mold; can cause opportunistic infections	More frequently isolated from oral cavity in elderly patients with compromised immune systems.	Weakened immune defenses in elderly lead to colonization and potential oral aspergillosis. Produces potent mycotoxins and proteases, contributing to tissue invasion and immune evasion.	Oral aspergillosis, rare but severe infections.	Deepa et al., 2014; Vieira et al., 2017; [37,38]
*Cladosporium species*	Common environmental mold; generally non-pathogenic	Detected more frequently in oral cavities of elderly individuals with poor oral hygiene.	Aging and compromised oral hygiene create environment conducive to colonization. Produces allergens and mycotoxins, which can exacerbate local inflammation and immune response.	Contributes to fungal dysbiosis, potential pathogenic implications.	Fechney et al., 2018 [59]

## 3. Morbidities Associated with Postmenopausal Status

The decline in endogenous estrogen levels during menopause increases the risk of various morbidities, including vasomotor symptoms, osteoporosis, cardiovascular diseases, malignancies, and dementia. These physiological changes and symptoms significantly impact the quality of life for postmenopausal women.

The morbidities associated with menopause include cardiovascular and cerebrovascular conditions, deep venous and pulmonary embolism, dyslipidemia, obesity, diabetes, dementia, breast and genital cancers, and osteoporosis-related fractures [60]. Additionally, sleep disorders [61,62] and urogenital disorders [63] are also prevalent during this stage.

Multiple morbidities often coexist, and medications prescribed for one condition may adversely affect others. For instance, treatments for postmenopausal osteoporosis can interact with other metabolic diseases [64]. Moreover, conditions like articular disorders or depression can impair oral hygiene due to difficulties in maintenance or neglect, thereby disrupting oral microbiome homeostasis.

These overlapping conditions complicate the management of the postmenopausal state and increase the risk of mortality. Women with multiple morbidities face a higher risk of all-cause mortality compared to premenopausal women [65].

Tooth loss is associated with postmenopausal osteoporosis; however, a recent study found that oral hygiene, rather than bone mineral density, is more closely linked to tooth loss [66], underscoring the importance of oral microbiota in maintaining oral health. Furthermore, dental treatments, including dental implants, are viable options for postmenopausal women with a stable general health status [67,68].

Osteoporosis is linked with important changes in gut bacteria, fungi, and fecal metabolites in postmenopausal women, and with patients’ bone mineral density, as demonstrated in a recent study [69].

Both menopause and aging are associated with an increased risk of morbidities, but the underlying mechanisms differ. In menopause, the primary mechanism is the significant drop in estrogen levels, which affects various physiological systems, leading to conditions such as osteoporosis, cardiovascular diseases, and metabolic disorders. In contrast, the mechanism associated with aging is cellular senescence, a process where cells lose the ability to divide and function properly, contributing to tissue degeneration and age-related diseases like cancer, Alzheimer’s disease, and frailty.

Additionally, there is substantial individual variability in the aging process between men and women, influenced by hormonal changes. Many age-related diseases exhibit sex-specific patterns due to mechanisms linked to sex chromosomes and hormonally driven differences [70]. Women tend to have higher levels of frailty, but men are more vulnerable to mortality [71].

The influence of sex on various disease processes indicates that aging is not driven by a single factor [72]; rather, multiple mechanisms interact in complex ways without a clear, uniform pattern.

Increasing evidence suggests that sex hormones not only influence lifespan but also interact with the immune system, mitochondrial function, telomere length, and gut microbiota [73]. These factors collectively regulate sex differences from embryonic development through aging, significantly impacting aging-related diseases.

A recent study has highlighted the critical role of sex and the gut microbiome in the development, progression, and treatment of cardiovascular disease [74], underscoring the importance of understanding the relationship between the microbiome, sex, and disease in treatment planning.

Postmenopausal status is associated not only with a variety of morbidities due to hormonal changes, but also with one troublesome oral condition, xerostomia. This oral condition is related to other systemic conditions and medications.

## 4. Systemic Conditions and Medication Linked to Xerostomia

Salivary secretion plays a vital role in maintaining oral health, and its impairment can lead to significant discomfort and an increased risk of oral diseases, particularly in the elderly population [50]. Aging is associated with a natural decline in salivary gland function, resulting in a condition known as xerostomia, or dry mouth [75]. This condition is not only prevalent but also particularly impactful in older adults, affecting their quality of life by impairing their ability to eat, speak, and maintain oral hygiene [76]. The decline in salivary secretion in elderly individuals is often compounded by the use of multiple medications, a common occurrence in this age group [77]. Many medications (Table 2), particularly those with anticholinergic properties, exacerbate salivary gland hypofunction, leading to a further decrease in saliva production [52].

**Table 2:** Medications and their impact on xerostomia in elderly individuals with dysbiosis.

Medication Class	Specific Medications	Mechanism of Action	Impact on Xerostomia in Elderly	References
Anticholinergics	Atropine, Scopolamine, Oxybutynin	Blocks acetylcholine receptors, reducing saliva production.	Significant reduction in salivary flow, leading to dry mouth.	Arany et al., 2021 [78]
Antidepressants	Tricyclic Antidepressants (e.g., Amitriptyline), SSRIs	Anticholinergic effects, serotonin reuptake inhibition.	Decreased saliva production, commonly associated with dry mouth in elderly.	Teoh et al., 2023 [79]
Antihypertensives	Beta-blockers (e.g., Propranolol), Diuretics	Adrenergic blockade, reduction in fluid retention.	Reduced salivary secretion, contributing to dry mouth symptoms.	Ramírez Martínez-Acitores et al., 2020 [80]
Antipsychotics	Haloperidol, Risperidone	Dopamine antagonism, anticholinergic effects.	High risk of dry mouth due to significant reduction in salivary flow.	Stroup et al., 2018 [81]
Antihistamines	Diphenhydramine, Loratadine	H1 receptor antagonism with anticholinergic effects.	Decreases saliva production, leading to xerostomia, particularly in older adults.	Scully et al., 2003 [82]
Diuretics	Hydrochlorothiazide, Furosemide	Promotes diuresis, reducing fluid volume in body.	Reduces salivary secretion, contributing to dry mouth.	Prasanthi et al., 2014 [83]
Bronchodilators	Ipratropium, Tiotropium	Anticholinergic bronchodilation.	May cause dry mouth due to reduced saliva production.	Marcott et al., 2020 [84]
Sedatives and Hypnotics	Benzodiazepines (e.g., Diazepam), Zolpidem	CNS depression with muscle relaxation and reduced salivary flow.	Frequently leads to dry mouth in elderly patients.	Tiisanoja et al., 2016 [85]
Antiemetics	Metoclopramide, Ondansetron	Dopamine receptor antagonism, serotonin receptor antagonism.	Moderate risk of dry mouth due to reduced salivary secretion.	Migirov et al., 2024 [86]
Opioid Analgesics	Morphine, Codeine	CNS depression, reduced autonomic function.	Decreased saliva production, often leading to xerostomia.	Mercadante et al., 2019 [87]
Antiparkinsonian Agents	Levodopa, Benztropine	Dopamine precursor, anticholinergic properties.	Contributes to dry mouth through decreased salivary flow.	Escobar et al., 2019 [88]
Antiepileptics	Phenytoin, Carbamazepine	CNS effects with reduction in autonomic salivary control.	Frequently causes dry mouth in elderly individuals.	Ghafoor et al., 2014 [89]
Chemotherapy Agents	Cyclophosphamide, Methotrexate	Cytotoxic effects on rapidly dividing cells, including salivary glands.	Severe xerostomia due to reduced saliva production and glandular damage.	Nathan et al., 2023 [90]
Muscle Relaxants	Baclofen, Cyclobenzaprine	CNS depression, reduced neurotransmission.	Significant reduction in saliva production, leading to xerostomia.	Talha et al., 2023 [91]

The autonomic nervous system, which controls salivary gland function, undergoes changes with age, with a noted decrease in parasympathetic activity, which is primarily responsible for stimulating saliva production [92]. Consequently, the saliva produced in the elderly is often of lower volume and higher viscosity, which not only reduces its effectiveness in lubricating the oral mucosa but also diminishes its protective functions, such as buffering acids and controlling microbial growth [93]. The measurement of salivary flow rates in the elderly often reveals a significant reduction in both unstimulated and stimulated saliva production, correlating with the subjective experience of dry mouth reported by many older adults [94]. This condition is further exacerbated by systemic diseases (Table 3), such as diabetes and Sjögren’s syndrome, which are more prevalent in the elderly and contribute to reduced salivary gland function [95,96,97,98].

**Table 3:** Diseases associated with xerostomia in elderly patients and their consequences.

Disease	Impact on Xerostomia	Consequences of Xerostomia	References
Diabetes Mellitus	Elevated blood glucose levels and dehydration exacerbate dry mouth symptoms	Increased risk of oral infections, poor glycemic control, increased dental caries, and oral discomfort	Rohani et al., 2019 [95]
Sjögren’s Syndrome	Autoimmune attack on moisture-producing glands leads to severe xerostomia	Severe oral discomfort, difficulty in swallowing, dental caries, oral infections, and potential for malnutrition	Mathews et al., 2008 [97]
Rheumatoid Arthritis	Often associated with secondary Sjögren’s syndrome, impairing salivary gland function	Increased risk of dental decay, oral infections, difficulty in chewing, and increased dental plaque accumulation	Mehdipour et al., 2022 [98]
Parkinson’s Disease	Autonomic control over salivation is affected; medications often exacerbate xerostomia	Difficulty swallowing, increased risk of aspiration pneumonia, speech difficulties, and increased risk of dental problems	Auffret et al., 2021 [99]
Alzheimer’s Disease	Cognitive decline and medication side effects contribute to dry mouth	Increased dental caries, poor oral hygiene, difficulty in eating, risk of malnutrition, and increased risk of oral infections	Kulkarni et al., 2023 [100]
Hypertension	Antihypertensive drugs induce dry mouth as side effect	Oral discomfort, risk of dental caries, periodontal disease, and potential for altered taste and nutritional deficiencies	Ramírez Martínez-Acitores et al., 2020 [80]
Chronic Kidney Disease (CKD)	Uremia and medication-induced reduction in salivary flow	Oral ulcers, metallic taste, increased risk of oral infections, and potential exacerbation of uremic symptoms	Oyetola et al., 2015 [101]
Depression/Anxiety	Anticholinergic properties of antidepressants and anxiolytics contribute to xerostomia	Poor oral hygiene, increased risk of caries, oral discomfort, social withdrawal, and decreased quality of life	Kisely et al., 2016 [102]
Chronic Obstructive Pulmonary Disease (COPD)	Use of inhaled bronchodilators reduces salivary secretion	Dry mouth, difficulty speaking, increased risk of oral infections, and potential for exacerbation of respiratory symptoms	Marcott et al., 2020 [84]
Cancer (Radiotherapy)	Radiotherapy for head and neck cancers damages salivary glands, leading to severe xerostomia	Severe mucositis, difficulty eating, extreme discomfort, tooth decay, and increased risk of infections in oral cavity	Albu et al., 2016; Sroussi et al., 2017; Enășescu, at al., 2021; Petrescu et al., 2010; [103,104,105,106,107]

## 5. Oral Mycobiome in Postmenopausal Women and Aged Population

Few studies have investigated the oral microbiome of postmenopausal women, and those that have primarily focused on bacterial load collected from subgingival plaque. For instance, Brennan et al. identified a prevalence of specific bacterial infections in a large group of postmenopausal women, finding associations with oral bone loss and obesity [108]. Another significant correlation was observed between osteoporosis, missing teeth, and the presence of periodontal pathogens [109]. Additionally, a study by LaMonte et al. found an association between specific oral bacteria and an increased risk of developing hypertension among postmenopausal women [110].

While the importance of the mycobiome is increasingly recognized in women’s health [43], the oral mycobiome remains understudied in this context.

There are several factors associated with advancing age that are known to favor oral fungal colonization. Among these, reduced salivary flow rates, a condition known as xerostomia, are particularly significant, creating an environment conducive to fungal overgrowth [111,112]. Additionally, the use of total or partial dentures further increases the risk of fungal colonization, particularly by *Candida* species, due to the creation of niches that can harbor fungal communities [113].

Interestingly, despite these age-related changes, a recent study has shown that the composition of the salivary microbiome, in terms of both bacterial families and genera, remains relatively stable throughout the menstrual cycle and into menopause [114]. This suggests that while certain environmental and physiological factors may predispose older adults to fungal colonization, the broader microbial ecosystem within the oral cavity may maintain a degree of resilience in response to hormonal fluctuations.

Table 4 summarizes the studies that revealed mycobiome changes with age. The presented studies mainly investigated the oral microbiome and had a limited focus on sex differences. Healthy individuals maintain a mycobiome similar to that of younger adults; dysbiosis and increased fungal load were linked to aging and frailty. Age-related changes in the mycobiome are associated with decreased salivary flow and increased susceptibility to oral diseases in the elderly.

The oral mycobiome plays a critical role in maintaining oral health. Dysbiosis—an imbalance between commensal and pathogenic species of fungi and bacteria—is the key factor in the etiopathogenesis of various oral diseases. In a healthy oral environment, fungi and bacteria coexist in a balanced relationship, contributing to oral homeostasis. However, when this balance is disturbed, it can lead to oral diseases such as caries, periodontitis, peri-implantitis, oral fungal infections, oral potentially malignant disorders, and oral cancer [8]. In postmenopausal women, changes like mucosal atrophy, reduced salivary secretion, chronic morbidities, and polypharmacy favor the shift from a balanced oral microbial community to one dominated by fungi.

**Table 4:** Mycobiome changes with aging.

Targeted Group	Mycobiome Changes	Influence on Oral and General Health	Reference
280 institutionalized and 61 non-institutionalized elderly people (without sex differentiations).	Oral colonization with yeasts was more frequently found in institutionalized elderly.	A significantly higher level of hyposalivation and oral yeast colonization and poorer dental status in the institutionalized group as compared with the non-institutionalized group of elderly people.	Glazar et al., 2016 [115]
307 males and 613 females with complete upper dentures were selected for study and divided into four age groups: #50 years, 51–60, 61–70, and 70 years.	Statistically significant relationship between intensity of yeast growth and gender.	The genera of *Candida* species and the frequency of yeast infection in denture wearers appear to be influenced by both age and gender.	Loster et al., 2016 [15]
75–99 years of age, community-dwelling, healthy elderly group.	Significant association between denture use and increased fungal load in saliva, especially in complete denture wearers.	The denture appliance restricts the cleansing action of the tongue and saliva, which are part of the host defense mechanism. Oral candidiasis has been associated with hyposalivation, which promotes an unhealthy oral environment.	Zakaria et al., 2017 [116]
Elderly living in nursing homes with diabetuss mellitus and non-diabetus mellitus and healthy control group. Between 68 and 101 years (without sex differentiations).	In saliva samples from present diabetic group, levels of phylum Bacteroidetes and genus *Alloprevotella* were decreased, while levels of genera *Actinomyces* and *Selenomonas* were increased.	An abundance of *Actinomyces* associated with diabetus mellitus.	Ogawa et al., 2017 [117]
Elderly living in nursing homes and healthy controls.	Oral samples obtained from present nursing home residents showed greater levels of *Selenomonas, Veillonella*, and *Haemophilus*, and lower levels of *Fusobacterium*.	Frailty is associated with oral microbiota formation and composition.	Ogawa et al., 2018 [118]
Own homes and rest homes of older people, 13 females and 7 males in each group, over 70 years (without sex differentiations).	Yeast species most frequently isolated in each sample type from both participant groups was *C. albicans* followed by *C. glabrata*. Seven yeast species were identified in samples from individuals living in their own homes: *C. albicans*, *C. glabrata*, *C. parapsilosis*, *C. lusitaniae*, *C. guilliermondii*, *Pichia fermentans,* and *Yarrowia lipolytica*. Only five yeast species (*C. albicans*, *C. glabrata*, *Saccharomyces cerevisi*, *C. dubliniensis,* and *C. tropicalis*) could be identified in rest-home participants.	Species with the potential to be drug resistant were present in both the rest home residents (*C. albicans*, *C. glabrata*, *C. dubliniensis*, and *C. tropicalis*) and people living in their own homes (*C. albicans*, *C. glabrata*, *C. parapsilosis*, *C. lusitaniae*, *C. guilliermondii*, and *Y. lipolytica*). A significant association between low salivary flow rate and a higher number of medications. We found that oral sites were more frequently colonized by yeast in rest-home participants than in people living in their own home and that the mean concentration of yeast in the saliva of rest-home participants was 23 times higher than that in non-rest home participants (the median was 45 times higher).	Thiyahuddin et al., 2019 [119]
19 healthy young adults (19 to 33 years), 24 healthy elderly adults (68 to 88 years), and 22 centenarians.	No significant difference among three age groups was detected for fungal communities in oral cavity and gut. Dominant fungi in oral cavity were *Malassezia*, *Candida*, and *Saccharomyces*.	The bacterial and fungal communities in the oral cavity did not display distinct age-related clustering in healthy subjects.	Wu et al., 2020 [120]
356 Dutch community-dwelling older adults (65–93 years).	*Candida albicans* abundance seemed to be associated with poor smell.	Older age, edentation, poor smell, and poor appetite were associated with lower alpha diversity (indicating the intra-individual microbial diversity) and explained a significant amount of beta diversity (indicating inter-individual dissimilarity in microbiota composition).	Fluitman et al., [121]
38–80 years old.	Younger participants were more likely to have similar microbiota composition, whereas older participants demonstrated wider difference.	Salivary microbiota was associated with age and frailty.	Wells et al., 2022 [122]
38 subjects (23 female and 15 male), with 19 elderly adults.Mean age of 61.5.	Analyzed diversity and richness of oral mycobiota of patients clinically diagnosed with oral thrush, follow-up of oral thrush patients after antifungal therapy, and healthy controls. Presence of *Candida* and *Candida albicans* were significantly associated with oral thrush status.	Older age, greater risk of malnutrition, antibiotic treatment, concurrent bacterial infection, cancer, chemotherapy, denture usage, hypertension, and xerostomia were factors significantly associated with the oral thrush group. Sex, ethnicity, antimicrobial wash, topical/inhalational corticosteroid, smoking, diabetes, dyslipidemia, and HIV were not significantly associated with oral thrush.	Karajacob et al., 2023 [123]
Three age groups: 20–40; 40–60; 60+ years.	Low bacterial diversity with aging. Commensal *Neisseria* had declined after age of 40. Opportunistic pathogens *Streptococcus anginosus* and *Gemella sanguinis* gradually rose with age.	Prone to disease formation in the oral cavity as well as in distant body sites.	Kazarina et al., 2023 [124]; Albu et al., 2019 [125]
35–70 years old.	Changes in microbiota with age. Abundance of *Veillonella* was reduced in both males and females, whereas increases in *Corynebacterium* appeared specific to males and *Aggregatibacter*, *Fusobacterium, Neisseria*, *Stomatobaculum*, and *Porphyromonas* specific to females.	Age and frailty are differentially associated with measures of microbial diversity and composition.	DeClerq et al., 2024 [126]
Elderly adults receiving community support and home care service.	High-density fungal population co-occurs with poor oral and systemic health status.	The dysbiosis of the bacterial community, and the overgrowth of non-*albicans Candida* species, might worsen oral and systemic health.	Asakawa et al., 2024 [127]

## 6. Association Between Aging, Oral Mycobiome Alterations and Inflammation

Aging and menopause disrupt the immune system and oral health, and when combined with environmental factors such as diet, medication, and oral hygiene, these changes influence the composition of the oral mycobiome. An altered oral mycobiome can significantly impact both oral and systemic health, often promoting inflammation. As mentioned earlier, oral dysbiosis and an increased fungal load are associated with frailty.

Frailty, strongly linked with aging, is a common clinical syndrome in older adults that increases the risk of poor health outcomes, including falls, disability, hospitalization, and mortality [128]. It represents a state of decreased physiological reserves, leading to heightened vulnerability to adverse outcomes when exposed to stressors [129]. These factors have significant clinical implications for the quality of life in postmenopausal women and may suggest the potential use of probiotics to restore oral homeostasis.

Chronic diseases and aging are associated with low-level systemic inflammation, as indicated by elevated levels of biomarkers such as high-sensitivity C-reactive protein (CRP), interleukin-6 (IL-6), interleukin-10 (IL-10), interleukin-1 receptor antagonist (IL-1RA), insulin-like growth factor 1 (IGF-1), and soluble tumor necrosis factor receptor 1 (sTNFR-1) [130]. These biomarkers tend to be elevated in the elderly population [131]. Recognized sources of chronic inflammation include aging, an unbalanced diet, low levels of sex hormones, and smoking [132,133].

Persistent low-grade inflammation, which is common in the elderly, contributes to the degeneration of multiple organs. There is strong evidence linking the development of age-related, multifactorial conditions—such as cancer, cardiovascular disease, Alzheimer’s disease, type II diabetes, frailty, sarcopenia, and osteoporosis—with low-grade elevations of circulating inflammatory mediators [134].

During aging, many changes occur in the oral cavity that can lead to chronic inflammation [135], and inflammatory networks connect the oral microbiome with systemic health and disease. A healthy, commensal oral microbiome is associated with a balanced state, while dysbiosis is linked to disease. The oral microbiome interacts with inflammation and the immune system, potentially affecting major organs and contributing to various illnesses.

Given that an increase in fungal load is associated with an impaired immune system [7,8], inflammation appears to be closely related to alterations in the oral mycobiome.

## 7. Suggestions for Future Research

While the current understanding of the oral mycobiome and its interactions with hormonal changes, particularly during menopause, has advanced significantly, several key areas warrant further investigation.

Future research should focus on longitudinal studies to elucidate the precise mechanisms by which hormonal fluctuations during menopause influence the composition and function of the oral mycobiome. This includes exploring the role of less-studied fungal species and their potential pathogenicity in postmenopausal women.

Additionally, the impact of various therapeutic interventions, such as hormone replacement therapy or probiotics, on the oral mycobiome and overall oral health should be assessed. Research should also consider the influence of systemic conditions and medications commonly associated with aging on oral mycobiome dynamics.

Finally, advancing molecular techniques, such as next-generation sequencing and metagenomics, could offer deeper insights into the complex interactions between the mycobiome, oral health, and systemic health, potentially leading to novel therapeutic approaches for managing oral dysbiosis in postmenopausal women.

## 8. The Limitations of the Study

To our knowledge, this is the first study to explore the relationship between the oral mycobiota, postmenopausal women, and inflammation. However, it is important to note that this review is descriptive in nature, and the findings highlighted here underscore the need for confirmation through longitudinal and large-scale studies to achieve a more comprehensive understanding.

## 9. Conclusions

This review highlights the complex relationship between the oral mycobiome and hormonal changes during menopause, emphasizing their potential impact on both oral and systemic health. The oral cavity hosts a diverse range of fungal species, with Candida being the most dominant. The composition of the oral mycobiome is significantly influenced by the hormonal shifts that occur during menopause. The reduction in estrogen levels contributes to dysbiosis, favoring the overgrowth of opportunistic fungal species, which may increase the risk of oral infections and contribute to systemic inflammation.

As our understanding of these processes continues to grow, there is hope that we will eventually be able to better address the negative outcomes associated with aging and menopause. Enhanced collaboration among healthcare providers from various specialties, with a comprehensive understanding of the aging process in postmenopausal women, will be crucial in improving therapeutic outcomes and the overall quality of life for these patients.

## Data Availability

Not applicable.
